# Supplementary material for: Psychometric Hepatic Encephalopathy Score for the Diagnosis of Minimal Hepatic Encephalopathy in Thai Cirrhotic Patients
Source: J Clin Med. 2023 Jan 8;12(2):519. doi: 10.3390/jcm12020519 (PMC9864758; doi:10.3390/jcm12020519)
Supplement: Supplementary file 1 [file jcm-12-00519-s001.zip › jcm-1996795-supplementary.pdf]

Supplement Table S1: Norm tables for the Thai population based on age and education level

1A	NCT-A	Primary School				
Score		1	0	-1	-2	-3
Age		Mean-1SD	Mean	Mean+1SD	Mean+2SD	Mean+3SD
18		23	33	46	65	92
19		23	33	46	65	92
20		23	33	47	66	93
21		24	33	47	67	94
22		24	34	48	67	95
23		24	34	48	68	96
24		24	34	49	69	97
25		25	35	49	69	98
26		25	35	49	70	99
27		25	35	50	70	100
28		25	36	50	71	100
29		25	36	51	72	101
30		26	36	51	72	102
31		26	37	52	73	103
32		26	37	52	74	104
33		26	37	53	74	105
34		27	38	53	75	106
35		27	38	54	76	107
36		27	38	54	77	108
37		27	39	55	77	109
38		28	39	55	78	110
39		28	39	56	79	111
40		28	40	56	79	112
41		28	40	57	80	113
42		29	41	57	81	114
43		29	41	58	82	115
44		29	41	58	82	116
45		30	42	59	83	117
46		30	42	59	84	119
47		30	42	60	85	120
48		30	43	61	86	121
49		31	43	61	86	122
50		31	44	62	87	123
51		31	44	62	88	124
52		31	44	63	89	125
53		32	45	63	90	126
54		32	45	64	90	128
55		32	46	65	91	129
56		33	46	65	92	130
57		33	47	66	93	131

58	33	47	66	94	132
59	34	47	67	95	134
60	34	48	68	95	135
61	34	48	68	96	136
62	35	49	69	97	137
63	35	49	70	98	139
64	35	50	70	99	140
65	35	50	71	100	141
66	36	51	71	101	143
67	36	51	72	102	144
68	36	52	73	103	145
69	37	52	73	104	147
70	37	52	74	105	148

1B	NCT-A	High School				
**Score**		1	0	-1	-2	-3
**Age**		**Mean-1SD**	**Mean**	**Mean+1SD**	**Mean+2SD**	**Mean+3SD**
18		19	27	38	53	76
19		19	27	38	54	76
20		19	27	39	54	77
21		19	28	39	55	78
22		20	28	39	55	78
23		20	28	40	56	79
24		20	28	40	56	80
25		20	29	40	57	81
26		20	29	41	58	81
27		21	29	41	58	82
28		21	29	41	59	83
29		21	30	42	59	84
30		21	30	42	60	84
31		21	30	43	60	85
32		22	30	43	61	86
33		22	31	43	61	87
34		22	31	44	62	87
35		22	31	44	63	88
36		22	32	45	63	89
37		23	32	45	64	90
38		23	32	45	64	91
39		23	33	46	65	92
40		23	33	46	65	92
41		23	33	47	66	93
42		24	33	47	67	94
43		24	34	48	67	95
44		24	34	48	68	96
45		24	34	49	69	97
46		25	35	49	69	98
47		25	35	49	70	99
48		25	35	50	70	100
49		25	36	50	71	100
50		25	36	51	72	101
51		26	36	51	72	102
52		26	37	52	73	103
53		26	37	52	74	104
54		26	37	53	74	105
55		27	38	53	75	106
56		27	38	54	76	107
57		27	38	54	77	108

58	27	39	55	77	109
59	28	39	55	78	110
60	28	39	56	79	111
61	28	40	56	79	112
62	28	40	57	80	113
63	29	41	57	81	114
64	29	41	58	82	115
65	29	41	58	82	116
66	30	42	59	83	117
67	30	42	59	84	119
68	30	42	60	85	120
69	30	43	61	86	121
70	31	43	61	86	122

1C	NCT-A	University				
Score		1	0	-1	-2	-3
**Age**		**Mean-1SD**	**Mean**	**Mean+1SD**	**Mean+2SD**	**Mean+3SD**
18		17	24	33	47	66
19		17	24	34	47	67
20		17	24	34	48	68
21		17	24	34	48	68
22		17	24	35	49	69
23		17	25	35	49	70
24		18	25	35	50	70
25		18	25	35	50	71
26		18	25	36	51	71
27		18	26	36	51	72
28		18	26	36	52	73
29		18	26	37	52	73
30		19	26	37	52	74
31		19	27	37	53	75
32		19	27	38	53	76
33		19	27	38	54	76
34		19	27	39	54	77
35		19	28	39	55	78
36		20	28	39	55	78
37		20	28	40	56	79
38		20	28	40	56	80
39		20	29	40	57	81
40		20	29	41	58	81
41		21	29	41	58	82
42		21	29	41	59	83
43		21	30	42	59	84
44		21	30	42	60	84
45		21	30	43	60	85
46		22	30	43	61	86
47		22	31	43	61	87
48		22	31	44	62	87
49		22	31	44	63	88
50		22	32	45	63	89
51		23	32	45	64	90
52		23	32	45	64	91
53		23	33	46	65	92
54		23	33	46	65	92
55		23	33	47	66	93
56		24	33	47	67	94
57		24	34	48	67	95

58	24	34	48	68	96
59	24	34	49	69	97
60	25	35	49	69	98
61	25	35	49	70	99
62	25	35	50	70	100
63	25	36	50	71	100
64	25	36	51	72	101
65	26	36	51	72	102
66	26	37	52	73	103
67	26	37	52	74	104
68	26	37	53	74	105
69	27	38	53	75	106
70	27	38	54	76	107

1D	NCT-B	Primary School				
Score		1	0	-1	-2	-3
Age		Mean-1SD	Mean	Mean+1SD	Mean+2SD	Mean+3SD
18		56	84	124	183	270
19		57	84	124	184	272
20		57	84	125	185	273
21		57	85	125	185	274
22		58	85	126	186	275
23		58	86	126	187	277
24		58	86	127	188	278
25		58	86	128	189	279
26		59	87	128	190	281
27		59	87	129	191	282
28		59	87	129	191	283
29		59	88	130	192	284
30		60	88	131	193	286
31		60	89	131	194	287
32		60	89	132	195	288
33		61	90	132	196	290
34		61	90	133	197	291
35		61	90	134	198	292
36		61	91	134	199	294
37		62	91	135	200	295
38		62	92	136	200	296
39		62	92	136	201	298
40		63	92	137	202	299
41		63	93	137	203	301
42		63	93	138	204	302
43		63	94	139	205	303
44		64	94	139	206	305
45		64	95	140	207	306
46		64	95	141	208	308
47		65	95	141	209	309
48		65	96	142	210	310
49		65	96	143	211	312
50		65	97	143	212	313
51		66	97	144	213	315
52		66	98	145	214	316
53		66	98	145	215	318
54		67	99	146	216	319
55		67	99	147	217	321
56		67	100	147	218	322
57		68	100	148	219	324

58	68	100	149	220	325
59	68	101	149	221	327
60	69	101	150	222	328
61	69	102	151	223	330
62	69	102	151	224	331
63	70	103	152	225	333
64	70	103	153	226	334
65	70	104	153	227	336
66	70	104	154	228	337
67	71	105	155	229	339
68	71	105	156	230	340
69	71	106	156	231	342
70	72	106	157	232	344

1E	NCT-B	High School				
Score	0	1		-1	-2	-3
Age		Mean-1SD	Mean	Mean+1SD	Mean+2SD	Mean+3SD
18		49	72	106	157	232
19		49	72	107	158	233
20		49	72	107	158	234
21		49	73	108	159	236
22		49	73	108	160	237
23		50	73	109	161	238
24		50	74	109	161	239
25		50	74	110	162	240
26		50	74	110	163	241
27		51	75	111	164	242
28		51	75	111	164	243
29		51	76	112	165	244
30		51	76	112	166	245
31		52	76	113	167	247
32		52	77	113	167	248
33		52	77	114	168	249
34		52	77	114	169	250
35		52	78	115	170	251
36		53	78	115	171	252
37		53	78	116	171	254
38		53	79	116	172	255
39		53	79	117	173	256
40		54	79	117	174	257
41		54	80	118	175	258
42		54	80	119	175	259
43		54	81	119	176	261
44		55	81	120	177	262
45		55	81	120	178	263
46		55	82	121	179	264
47		55	82	121	179	265
48		56	82	122	180	267
49		56	83	122	181	268
50		56	83	123	182	269
51		56	84	124	183	270
52		57	84	124	184	272
53		57	84	125	185	273
54		57	85	125	185	274
55		58	85	126	186	275
56		58	86	126	187	277
57		58	86	127	188	278

58	58	86	128	189	279
59	59	87	128	190	281
60	59	87	129	191	282
61	59	87	129	191	283
62	59	88	130	192	284
63	60	88	131	193	286
64	60	89	131	194	287
65	60	89	132	195	288
66	61	90	132	196	290
67	61	90	133	197	291
68	61	90	134	198	292
69	61	91	134	199	294
70	62	91	135	200	295

1F	NCT-B	University				
Score		1	0	-1	-2	-3
Age		Mean-1SD	Mean	Mean+1SD	Mean+2SD	Mean+3SD
18		44	65	96	142	210
19		44	65	96	143	211
20		44	65	97	143	212
21		44	66	97	144	213
22		45	66	98	145	214
23		45	66	98	145	215
24		45	67	99	146	216
25		45	67	99	147	217
26		45	67	100	147	218
27		46	68	100	148	219
28		46	68	100	149	220
29		46	68	101	149	221
30		46	69	101	150	222
31		47	69	102	151	223
32		47	69	102	151	224
33		47	70	103	152	225
34		47	70	103	153	226
35		47	70	104	153	227
36		48	70	104	154	228
37		48	71	105	155	229
38		48	71	105	156	230
39		48	71	106	156	231
40		49	72	106	157	232
41		49	72	107	158	233
42		49	72	107	158	234
43		49	73	108	159	236
44		49	73	108	160	237
45		50	73	109	161	238
46		50	74	109	161	239
47		50	74	110	162	240
48		50	74	110	163	241
49		51	75	111	164	242
50		51	75	111	164	243
51		51	76	112	165	244
52		51	76	112	166	245
53		52	76	113	167	247
54		52	77	113	167	248
55		52	77	114	168	249
56		52	77	114	169	250
57		52	78	115	170	251

58	53	78	115	171	252
59	53	78	116	171	254
60	53	79	116	172	255
61	53	79	117	173	256
62	54	79	117	174	257
63	54	80	118	175	258
64	54	80	119	175	259
65	54	81	119	176	261
66	55	81	120	177	262
67	55	81	120	178	263
68	55	82	121	179	264
69	55	82	121	179	265
70	56	82	122	180	267

1G	SDT	Primary School				
Score		1	0	-1	-2	-3
Age		Mean-1SD	Mean	Mean+1SD	Mean+2SD	Mean+3SD
**18**		53	76	107	151	213
**19**		54	76	107	151	214
**20**		54	76	108	152	215
**21**		54	77	108	153	216
**22**		54	77	109	153	217
**23**		55	77	109	154	218
**24**		55	78	110	155	219
**25**		55	78	110	156	220
**26**		55	78	111	156	221
**27**		56	79	111	157	222
**28**		56	79	112	158	223
**29**		56	79	112	158	224
**30**		56	80	113	159	225
**31**		57	80	113	160	226
**32**		57	81	114	161	227
**33**		57	81	114	161	228
**34**		58	81	115	162	229
**35**		58	82	115	163	230
**36**		58	82	116	164	231
**37**		58	82	116	164	232
**38**		59	83	117	165	233
**39**		59	83	117	166	234
**40**		59	84	118	167	236
**41**		59	84	119	167	237
**42**		60	84	119	168	238
**43**		60	85	120	169	239
**44**		60	85	120	170	240
**45**		61	86	121	171	241
**46**		61	86	121	171	242
**47**		61	86	122	172	243
**48**		61	87	122	173	244
**49**		62	87	123	174	245
**50**		62	87	124	175	247
**51**		62	88	124	175	248
**52**		63	88	125	176	249
**53**		63	89	125	177	250
**54**		63	89	126	178	251
**55**		63	90	126	179	252
**56**		64	90	127	179	254
**57**		64	90	128	180	255

**58**	64	91	128	181	256
**59**	65	91	129	182	257
**60**	65	92	129	183	258
**61**	65	92	130	184	259
**62**	65	92	131	185	261
**63**	66	93	131	185	262
**64**	66	93	132	186	263
**65**	66	94	132	187	264
**66**	67	94	133	188	265
**67**	67	95	134	189	267
**68**	67	95	134	190	268
**69**	68	95	135	191	269
**70**	68	96	136	191	270

1H	SDT	High School				
**Score**		1	0	-1	-2	-3
**Age**		**Mean-1SD**	**Mean**	**Mean+1SD**	**Mean+2SD**	**Mean+3SD**
**18**		44	62	88	124	175
**19**		44	63	88	125	176
**20**		44	63	89	125	177
**21**		45	63	89	126	178
**22**		45	63	90	126	179
**23**		45	64	90	127	179
**24**		45	64	90	128	180
**25**		45	64	91	128	181
**26**		46	65	91	129	182
**27**		46	65	92	129	183
**28**		46	65	92	130	184
**29**		46	65	92	131	185
**30**		47	66	93	131	185
**31**		47	66	93	132	186
**32**		47	66	94	132	187
**33**		47	67	94	133	188
**34**		47	67	95	134	189
**35**		48	67	95	134	190
**36**		48	68	95	135	191
**37**		48	68	96	136	191
**38**		48	68	96	136	192
**39**		49	69	97	137	193
**40**		49	69	97	137	194
**41**		49	69	98	138	195
**42**		49	70	98	139	196
**43**		49	70	99	139	197
**44**		50	70	99	140	198
**45**		50	70	100	141	199
**46**		50	71	100	141	200
**47**		50	71	100	142	200
**48**		51	71	101	143	201
**49**		51	72	101	143	202
**50**		51	72	102	144	203
**51**		51	72	102	145	204
**52**		52	73	103	145	205
**53**		52	73	103	146	206
**54**		52	73	104	147	207
**55**		52	74	104	147	208
**56**		52	74	105	148	209
**57**		53	74	105	149	210

**58**	53	75	106	149	211
**59**	53	75	106	150	212
**60**	53	76	107	151	213
**61**	54	76	107	151	214
**62**	54	76	108	152	215
**63**	54	77	108	153	216
**64**	54	77	109	153	217
**65**	55	77	109	154	218
**66**	55	78	110	155	219
**67**	55	78	110	156	220
**68**	55	78	111	156	221
**69**	56	79	111	157	222
**70**	56	79	112	158	223

1I	SDT	University				
Score		1	0	-1	-2	-3
Age		Mean-1SD	Mean	Mean+1SD	Mean+2SD	Mean+3SD
**18**		39	55	77	109	154
**19**		39	55	78	110	155
**20**		39	55	78	110	156
**21**		39	55	78	111	156
**22**		39	56	79	111	157
**23**		40	56	79	112	158
**24**		40	56	79	112	158
**25**		40	56	80	113	159
**26**		40	57	80	113	160
**27**		40	57	81	114	161
**28**		41	57	81	114	161
**29**		41	58	81	115	162
**30**		41	58	82	115	163
**31**		41	58	82	116	164
**32**		41	58	82	116	164
**33**		41	59	83	117	165
**34**		42	59	83	117	166
**35**		42	59	84	118	167
**36**		42	59	84	119	167
**37**		42	60	84	119	168
**38**		42	60	85	120	169
**39**		43	60	85	120	170
**40**		43	61	86	121	171
**41**		43	61	86	121	171
**42**		43	61	86	122	172
**43**		43	61	87	122	173
**44**		44	62	87	123	174
**45**		44	62	87	124	175
**46**		44	62	88	124	175
**47**		44	63	88	125	176
**48**		44	63	89	125	177
**49**		45	63	89	126	178
**50**		45	63	90	126	179
**51**		45	64	90	127	179
**52**		45	64	90	128	180
**53**		45	64	91	128	181
**54**		46	65	91	129	182
**55**		46	65	92	129	183
**56**		46	65	92	130	184
**57**		46	65	92	131	185

**58**	47	66	93	131	185
**59**	47	66	93	132	186
**60**	47	66	94	132	187
**61**	47	67	94	133	188
**62**	47	67	95	134	189
**63**	48	67	95	134	190
**64**	48	68	95	135	191
**65**	48	68	96	136	191
**66**	48	68	96	136	192
**67**	49	69	97	137	193
**68**	49	69	97	137	194
**69**	49	69	98	138	195
**70**	49	70	98	139	196

1J	LTTsum	Primary School				
Score		1	0	-1	-2	-3
Age		Mean-1SD	Mean	Mean+1SD	Mean+2SD	Mean+3SD
18		80	110	154	220	324
19		81	110	154	221	325
20		81	111	155	222	327
21		81	111	155	223	328
22		81	111	156	224	330
23		82	112	157	225	331
24		82	112	157	226	332
25		82	113	158	226	334
26		82	113	158	227	335
27		83	113	159	228	337
28		83	114	159	229	338
29		83	114	160	230	340
30		83	114	161	231	341
31		84	115	161	232	343
32		84	115	162	233	344
33		84	116	163	234	345
34		85	116	163	235	347
35		85	117	164	236	348
36		85	117	164	237	350
37		85	117	165	238	351
38		86	118	166	239	353
39		86	118	166	240	355
40		86	119	167	241	356
41		86	119	167	242	358
42		87	119	168	243	359
43		87	120	169	243	361
44		87	120	169	244	362
45		88	121	170	245	364
46		88	121	171	246	365
47		88	122	171	247	367
48		89	122	172	248	369
49		89	122	173	249	370
50		89	123	173	251	372
51		89	123	174	252	373
52		90	124	175	253	375
53		90	124	175	254	377
54		90	125	176	255	378
55		91	125	177	256	380
56		91	125	177	257	382
57		91	126	178	258	383

**58**	91	126	179	259	385
**59**	92	127	179	260	387
**60**	92	127	180	261	389
**61**	92	128	181	262	390
**62**	93	128	181	263	392
**63**	93	129	182	264	394
**64**	93	129	183	265	395
**65**	94	130	183	266	397
**66**	94	130	184	267	399
**67**	94	131	185	269	401
**68**	95	131	186	270	402
**69**	95	131	186	271	404
**70**	95	132	187	272	406

1K	LTTsum	High School				
Score		1	0	-1	-2	-3
Age		Mean-1SD	Mean	Mean+1SD	Mean+2SD	Mean+3SD
18		76	103	143	205	299
19		76	103	144	205	301
20		76	104	144	206	302
21		76	104	145	207	303
22		77	104	146	208	304
23		77	105	146	209	306
24		77	105	147	209	307
25		77	105	147	210	308
26		78	106	148	211	310
27		78	106	148	212	311
28		78	107	149	213	312
29		78	107	149	214	313
30		79	107	150	214	315
31		79	108	150	215	316
32		79	108	151	216	317
33		79	108	152	217	319
34		80	109	152	218	320
35		80	109	153	219	322
36		80	110	153	220	323
37		80	110	154	220	324
38		81	110	154	221	326
39		81	111	155	222	327
40		81	111	156	223	328
41		81	111	156	224	330
42		82	112	157	225	331
43		82	112	157	226	333
44		82	113	158	227	334
45		82	113	158	228	336
46		83	113	159	229	337
47		83	114	160	229	338
48		83	114	160	230	340
49		83	115	161	231	341
50		84	115	161	232	343
51		84	115	162	233	344
52		84	116	163	234	346
53		85	116	163	235	347
54		85	117	164	236	349
55		85	117	164	237	350
56		85	117	165	238	352
57		86	118	166	239	353

**58**	86	118	166	240	355
**59**	86	119	167	241	356
**60**	87	119	168	242	358
**61**	87	120	168	243	360
**62**	87	120	169	244	361
**63**	87	120	170	245	363
**64**	88	121	170	246	364
**65**	88	121	171	247	366
**66**	88	122	171	248	367
**67**	89	122	172	249	369
**68**	89	123	173	250	371
**69**	89	123	173	251	372
**70**	89	123	174	252	374

1L	LTTsum	University				
Score		1	0	-1	-2	-3
Age		Mean-1SD	Mean	Mean+1SD	Mean+2SD	Mean+3SD
18		73	99	137	195	284
19		73	99	138	196	285
20		73	99	138	196	286
21		73	100	139	197	288
22		74	100	139	198	289
23		74	100	140	199	290
24		74	101	140	199	291
25		74	101	141	200	292
26		74	101	141	201	294
27		75	102	142	202	295
28		75	102	142	203	296
29		75	102	143	203	297
30		75	103	143	204	299
31		76	103	144	205	300
32		76	103	144	206	301
33		76	104	145	207	302
34		76	104	145	207	304
35		77	105	146	208	305
36		77	105	146	209	306
37		77	105	147	210	308
38		77	106	147	211	309
39		78	106	148	211	310
40		78	106	148	212	311
41		78	107	149	213	313
42		78	107	150	214	314
43		79	107	150	215	315
44		79	108	151	216	317
45		79	108	151	217	318
46		79	109	152	217	320
47		80	109	152	218	321
48		80	109	153	219	322
49		80	110	154	220	324
50		80	110	154	221	325
51		81	110	155	222	326
52		81	111	155	223	328
53		81	111	156	224	329
54		81	112	156	224	331
55		82	112	157	225	332
56		82	112	158	226	333
57		82	113	158	227	335

**58**	83	113	159	228	336
**59**	83	114	159	229	338
**60**	83	114	160	230	339
**61**	83	114	161	231	341
**62**	84	115	161	232	342
**63**	84	115	162	233	344
**64**	84	116	162	234	345
**65**	84	116	163	235	347
**66**	85	116	164	236	348
**67**	85	117	164	236	350
**68**	85	117	165	237	351
**69**	86	118	165	238	353
**70**	86	118	166	239	354

1M	DST	Primary School				
Score		**1**	0	-1	**-2**	**-3**
Age		Mean+1SD	Mean	Mean-1SD	Mean-2SD	Mean-3SD
18		59	49	39	30	20
19		58	49	39	29	20
20		58	48	38	29	19
21		57	48	38	28	19
22		57	47	38	28	18
23		56	47	37	27	18
24		56	46	37	27	17
25		55	46	36	26	17
26		55	45	36	26	16
27		54	45	35	25	16
28		54	44	35	25	15
29		53	44	34	24	15
30		53	43	34	24	14
31		52	43	33	23	14
32		52	42	33	23	13
33		51	42	32	22	13
34		51	41	32	22	12
35		51	41	31	21	12
36		50	40	31	21	11
37		50	40	30	20	11
38		49	39	30	20	10
39		49	39	29	19	10
40		48	38	29	19	9
41		48	38	28	18	9
42		47	37	28	18	8
43		47	37	27	17	8
44		46	36	27	17	7
45		46	36	26	16	7
46		45	35	26	16	6
47		45	35	25	16	6
48		44	34	25	15	5
49		44	34	24	15	5
50		43	33	24	14	4
51		43	33	23	14	4
52		42	32	23	13	3
53		42	32	22	13	3
54		41	31	22	12	2
55		41	31	21	12	2
56		40	30	21	11	1
57		40	30	20	11	1
58		39	29	20	10	0

59	39	29	19	10	0
60	38	29	19	9	0
61	38	28	18	9	0
62	37	28	18	8	0
63	37	27	17	8	0
64	36	27	17	7	0
65	36	26	16	7	0
66	35	26	16	6	0
67	35	25	15	6	0
68	34	25	15	5	0
69	34	24	14	5	0
70	33	24	14	4	0

1N	DST	High school				
Score		**1**	0	-1	**-2**	**-3**
**Age**		**Mean+1SD**	**Mean**	**Mean-1SD**	**Mean-2SD**	**Mean-3SD**
18		67	58	48	38	29
19		67	57	47	38	28
20		66	57	47	37	28
21		66	56	46	37	27
22		65	56	46	36	27
23		65	55	45	36	26
24		64	55	45	35	26
25		64	54	45	35	25
26		63	54	44	34	25
27		63	53	44	34	24
28		62	53	43	33	24
29		62	52	43	33	23
30		61	52	42	32	23
31		61	51	42	32	22
32		60	51	41	31	22
33		60	50	41	31	21
34		59	50	40	30	21
35		59	49	40	30	20
36		58	49	39	29	20
37		58	48	39	29	19
38		58	48	38	28	19
39		57	47	38	28	18
40		57	47	37	27	18
41		56	46	37	27	17
42		56	46	36	26	17
43		55	45	36	26	16
44		55	45	35	25	16
45		54	44	35	25	15
46		54	44	34	24	15
47		53	43	34	24	14
48		53	43	33	23	14
49		52	42	33	23	13
50		52	42	32	23	13
51		51	41	32	22	12
52		51	41	31	22	12
53		50	40	31	21	11
54		50	40	30	21	11
55		49	39	30	20	10
56		49	39	29	20	10
57		48	38	29	19	9
58		48	38	28	19	9

59	47	37	28	18	8
60	47	37	27	18	8
61	46	36	27	17	7
62	46	36	26	17	7
63	45	36	26	16	6
64	45	35	25	16	6
65	44	35	25	15	5
66	44	34	24	15	5
67	43	34	24	14	4
68	43	33	23	14	4
69	42	33	23	13	3
70	42	32	22	13	3

1O DST University

Score	1	0	-1	-2	-3
Age	Mean+1SD	Mean	Mean-1SD	Mean-2SD	Mean-3SD
18	73	63	54	44	34
19	73	63	53	43	34
20	72	62	53	43	33
21	72	62	52	42	33
22	71	61	52	42	32
23	71	61	51	41	32
24	70	60	51	41	31
25	70	60	50	40	31
26	69	59	50	40	30
27	69	59	49	39	30
28	68	58	49	39	29
29	68	58	48	38	29
30	67	57	48	38	28
31	67	57	47	38	28
32	66	56	47	37	27
33	66	56	46	37	27
34	65	55	46	36	26
35	65	55	45	36	26
36	64	54	45	35	25
37	64	54	44	35	25
38	63	53	44	34	24
39	63	53	43	34	24
40	62	52	43	33	23
41	62	52	42	33	23
42	61	51	42	32	22
43	61	51	41	32	22
44	60	51	41	31	21
45	60	50	40	31	21
46	59	50	40	30	20
47	59	49	39	30	20
48	58	49	39	29	19
49	58	48	38	29	19
50	57	48	38	28	18
51	57	47	37	28	18
52	56	47	37	27	17
53	56	46	36	27	17
54	55	46	36	26	16
55	55	45	35	26	16
56	54	45	35	25	16
57	54	44	34	25	15
58	53	44	34	24	15
59	53	43	33	24	14
60	52	43	33	23	14

**61**	52	42	32	23	13
**62**	51	42	32	22	13
**63**	51	41	31	22	12
**64**	50	41	31	21	12
**65**	50	40	30	21	11
**66**	49	40	30	20	11
**67**	49	39	29	20	10
**68**	48	39	29	19	10
**69**	48	38	29	19	9
**70**	47	38	28	18	9

Supplementary Table S2 The analysis of Youden's index with the different cut-offs of the simplified Animal Naming Test

S-ANT1 cut-off	Sensitivity	Specificity	Youden's index
< 10	5.8	99.3	0.051
< 11	7.7	99.3	0.07
< 12	7.7	97.9	0.056
< 13	13.5	97.9	0.114
< 14	15.4	97.2	0.126
< 15	25	96.5	0.215
< 16	32.7	95.1	0.278
< 17	36.5	93	0.295
< 18	48.1	86	0.341
< 19	50	80.4	0.304
< 20	57.7	76.9	0.346
< 21	63.5	69.2	0.327
**< 22**	**71.2**	**65**	**0.362**
< 23	76.9	58.7	0.356
< 24	80.8	55.2	0.36
< 25	82.7	49	0.317
< 26	84.6	41.3	0.259
< 27	90.4	35.7	0.261
< 28	90.4	30.1	0.205
< 29	92.3	28	0.203
< 30	94.2	23.1	0.173
